# Role of stromal PD-L1 expression in colorectal liver metastasis

**DOI:** 10.1186/s12885-024-11869-8

**Published:** 2024-01-17

**Authors:** Chie Takasu, Yuji Morine, Kozo Yoshikawa, Toshihiro Nakao, Takuya Tokunaga, Masaaki Nishi, Hideya Kashihara, Yuma Wada, Toshiaki Yoshimoto, Mitsuo Shimada

**Affiliations:** Department of Surgery, Tokushima University, Tokushima, Japan

**Keywords:** Immune check point, Stroma, PD-1

## Abstract

**Background and Aim:**

The outcomes of immune checkpoint blockade for colorectal cancer (CRC) treatment are unsatisfactory. Furthermore, the efficacy of immune checkpoint blockade for liver metastasis of various cancer is poor. Here, we investigated the relationship between stromal programmed death-ligand 1 (PD-L1) expression and the prognosis of patients with colorectal cancer liver metastasis (CRLM).

**Methods:**

The present study enrolled 84 CRLM patients who underwent surgery (R0) for CRC. Immunohistochemistry was performed to analyze stromal PD-L1 expression in CRLM.

**Results:**

Stromal PD-L1 was expressed in 52.3% of CRLM samples, which was associated with fewer not optimally resectable metastases (*p =* 0.04). Stromal PD-L1 also tended to associate with a lower tumor grade (*p* = 0.08). Stromal PD-L1-positive patients had longer overall survival (*p =* 0.003). Multivariate analysis identified stromal PD-L1 expression (*p =* 0.008) and poorer differentiation (*p* < 0.001) as independent prognostic indicators. Furthermore, stromal PD-L1 expression was correlated to a high number of tumor-infiltrating lymphocytes (TILs). Stromal PD-L1– and low TIL groups had shorter OS than stromal PD-L1 + and high TIL groups (46.6% vs. 81.8%, *p =* 0.05) Stromal PD-L1-positive patients had longer disease-free survival (DFS) (*p =* 0.03) and time to surgical failure (*p =* 0.001). Interestingly, stromal PD-L1 expression was positively related to the desmoplastic subtype (*p =* 0.0002) and inversely related to the replacement subtype of the histological growth pattern (*p =* 0.008).

**Conclusions:**

Stromal PD-L1 expression may be a significant prognostic marker for CRLM.

**Supplementary Information:**

The online version contains supplementary material available at 10.1186/s12885-024-11869-8.

## Introduction

Colorectal cancer (CRC) is steadily increasing and has the second highest incidence among all cancers worldwide [1]. Approximately 25% of CRC patients have liver metastasis at the time of diagnosis. Moreover, approximately 50% of patients develop liver metastasis during disease progression [2]. Therefore, treatment of CRC liver metastasis (CRLM) is important to improve the prognosis of CRC patients.

Recent research has focused on personalized treatments on the basis of immunotherapy by employing checkpoint blockade and immunomodulatory antibodies. Expression of programmed death-1 (PD-1) ligand 1 (PD-L1), predicts the effect of immune checkpoint blockade on gastric cancer [3]. However, the utility of PD-L1 expression as a prognostic and predictive factor of CRC remains controversial [3]. Previous studies have shown that PD-L1 expression in CRC is correlated to both better survival [4, 5] and poor survival [6–8]. Furthermore, the efficacy of immune blockade for liver metastasis of various cancers including CRC is unsatisfactory [9–11]. We previously reported the characteristics of immune markers in CRLM including PD-1, PD-L1, tumor-associated macrophages and indoleamine-pyrrole 2,3-dioxygenase [12]. Expression of these immunosuppressive proteins correlates to favorable survival and diminished tumor aggressiveness of CRLM. PD-L1 expression in CRLM is significantly correlated to better differentiation and low incidence of lymph node metastasis, which contribute to better overall survival.

Stromal cells in the tumor microenvironment have been the focus of recent studies [13, 14]. PD-L1 is primarily detected on tumor cells and overexpressed in various tumor cell types but also expressed in stromal cells such as lymphocytes, macrophages, dendritic cells, and fibroblasts [15]. Regarding PD-L1 expression in stromal cells, there have been some studies of small cohorts, but its role in prognosis is controversial, even in the primary site of CRC [16–18]. Only one previous study analyzed the expression patterns of stromal PD-L1 in the primary site and CRLM [19]. Stromal PD-L1 expression was higher than in the primary site in patients with synchronous metastasis, but its prognostic value was not elucidated. Furthermore, one study of stromal PD-L1 expression focused on a limited number of patients with colorectal liver oligometastasis defined as no more than five liver metastases [20]. Therefore, we evaluated the influence of stromal PD-L1 expression on CRLM and its prognostic value.

## Methods

### Patients

The present study enrolled 84 patients with CRLM who underwent surgery (R0) between 1995 and 2014 at Tokushima University Hospital. The classifications of liver metastasis, such as H-stage and grade, were defined in accordance with the Japanese Classification of Colorectal Carcinoma, Second English Edition [21]. Briefly, H-stage was classified by the number and maximum diameter of liver tumors. Grade classifications were determined by H-stage, mesenteric lymph node metastasis, and extrahepatic metastasis. The definition of non-optimally resectable was determined by previous reports as follows: single nodule (> 5 cm); multiple nodules (> 4 cm) and/or bilobar lesions; synchronous liver metastasis [22]. This study was reviewed and approved by the Institutional Review Board of the University of Tokushima Graduate School (Approval no. 2395, authorized in 2015). Written informed consent for inclusion was obtained from each patient. All methods were carried out in accordance with Declaration of Helsink.

### Histological assessment

Hematoxylin and eosin-stained tissue sections were used to evaluate tumor-infiltrating lymphocytes (TILs) that were quantitated in accordance with the International TIL Working Group 2014 Guidelines [23]. The cutoff value used to determine high and low TIL groups was defined in accordance with published data [24].

The histological characteristics of tumor growth pattern in CRC was evaluated with hematoxylin and eosin-stained tissue sections. Growth patterns classified as pushing, replacement, and desmoplastic [25]. The replacement (invasive) subtype is difficult to clearly distinguish at the tumor border. Tumor cells directly contact the liver parenchyma and replace hepatocytes and the hepatic sinusoidal structure. The desmoplastic subtype showed a tumor separates from the surrounding liver parenchyma by desmoplastic stroma formation [43, 44]. The pushing subtype showed expansive growth patterns. The tumor has clear border but no fibrotic tissue intervenes.

### Immunohistochemistry

Formalin-fixed, paraffin-embedded tissues were prepared for immunohistochemistry as described previously [12]. The paraffin-embedded samples were serially cut into 5-µm-thick sections that were dewaxed in xylene, rehydrated, and rinsed in a series of decreasing alcohol concentrations. Heat-induced antigen retrieval was performed in citrate buffer (pH 6.0) for 20 min in a microwave oven. The sections were incubated with Protein Block Serum-Free Reagent (DAKO, Carpinteria, CA, USA) for 30 min. Sections were incubated with a primary antibody at 4 °C overnight. Primary antibodies against the following proteins were used: PD-1 (AF1086, 1:40; R&D Systems, Minneapolis, MN, USA), PD-L1 (ab174838, 1:100; Abcam, Cambridge, UK), CD3 (ab5690, 1:100; Abcam), CD4 (ab125711, 1:100; Abcam), CD 8(M7103: Dako Corporation), CD68 (ab955, 1:100; Abcam), and αSMA (ab7817, 1:100; Abcam). Then, the sections were treated with secondary antibodies for 60 min. A Histofine SAB-PO Kit (Nichirei Biosciences Inc., Tokyo, Japan) was used for PD-1. An EnVision Dual Link System-HRP (K4065: Dako Corporation) was used for PD-L1, CD3, CD4, CD8, CD68, and αSMA. Finally, the sections were treated with 3,3-diaminobenzidine and counterstained with Mayer’s hematoxylin.

**Fig. 1:**
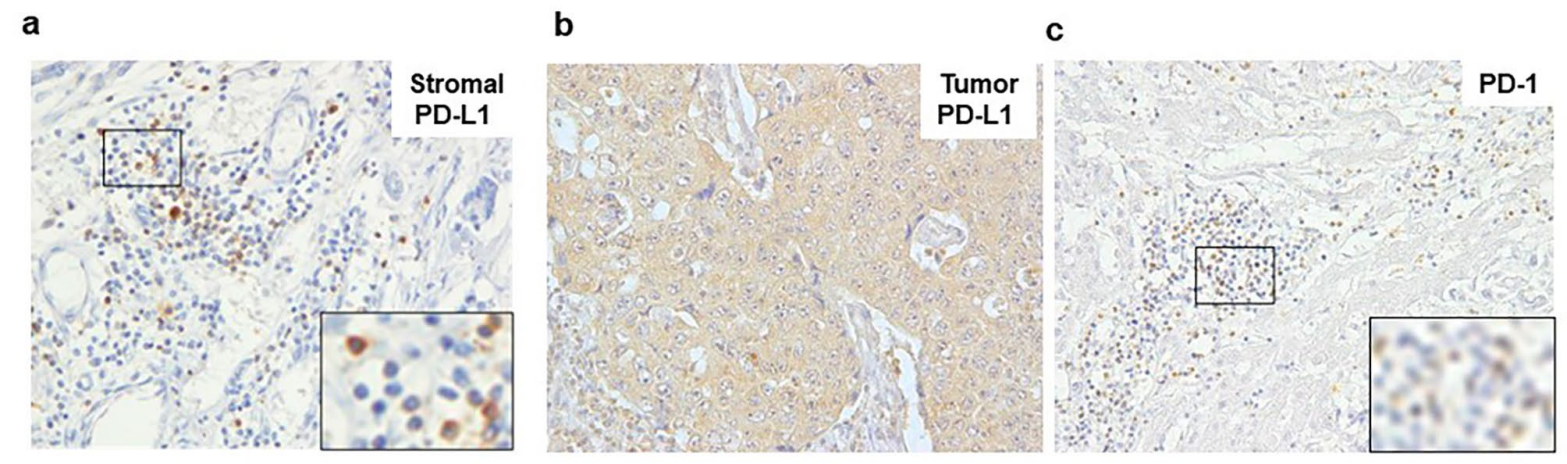
Representative IHC images. (**a**) Stromal PD-L1. (**b**) Tumor PD-L1. (**c**) PD-1 Magnification of the boxed area (black) is shown in the insets

PD-L1 expression was predominant in cytoplasm (Fig. 1a and b). The IHC score for PD-L1 expression in both stromal and tumor cells was calculated by the sum scoring of staining intensity and the distribution as described previously [12]. Briefly, staining intensity was scored as: 0, no staining; 1, weak; 2, moderate; 3, strong staining. Distribution was scored as follows, 0, ≤ 5%; 1, 6–25%; 2, 26–50%; 3, 51–75%; 4, 76–100%. Final score of more than 3 was defined as positive for PD-L1 expression in stromal cells [26, 27] and tumor cells [12, 28]. PD-1 expression was positive when > 40% of mononuclear cells in tumor tissues stained at ×400 magnification (Fig. 1c) [12, 29]. PD-1 positivity was defined as more than 40% of mononuclear cells were stained in the tumor at high power field of ×400. For the multiple liver metastases, the average score for every region was applied for evaluation.

### Statistical analysis

JMP 8.0.1 software (SAS, Cary, NC, USA) was used for statistical analysis. The statistical method to evaluate the relationship between two groups was the chi-squared test. Continuous variables are presented as the median and were compared by the Mann–Whitney test. Survival was calculated by Kaplan–Meier analysis and compared using the log-rank test. Multivariable Cox regression analysis was performed for variables with *p* < 0.05 in the univariate analysis. Time to surgical failure (TSF) was defined as the time from the initial curative surgery to an unresectable recurrence. Overall survival (OS) was defined as the time from curative surgery to death [30]. Because CRLM has unique biological characteristics, in which the first recurrence after an initial hepatic resection does not reflect surgical failure, TSF is a suitable endpoint for CRLM [30, 31]. *P* < 0.05 was considered statistically significant.

## Results

### Pathological findings

**Fig. 2:**
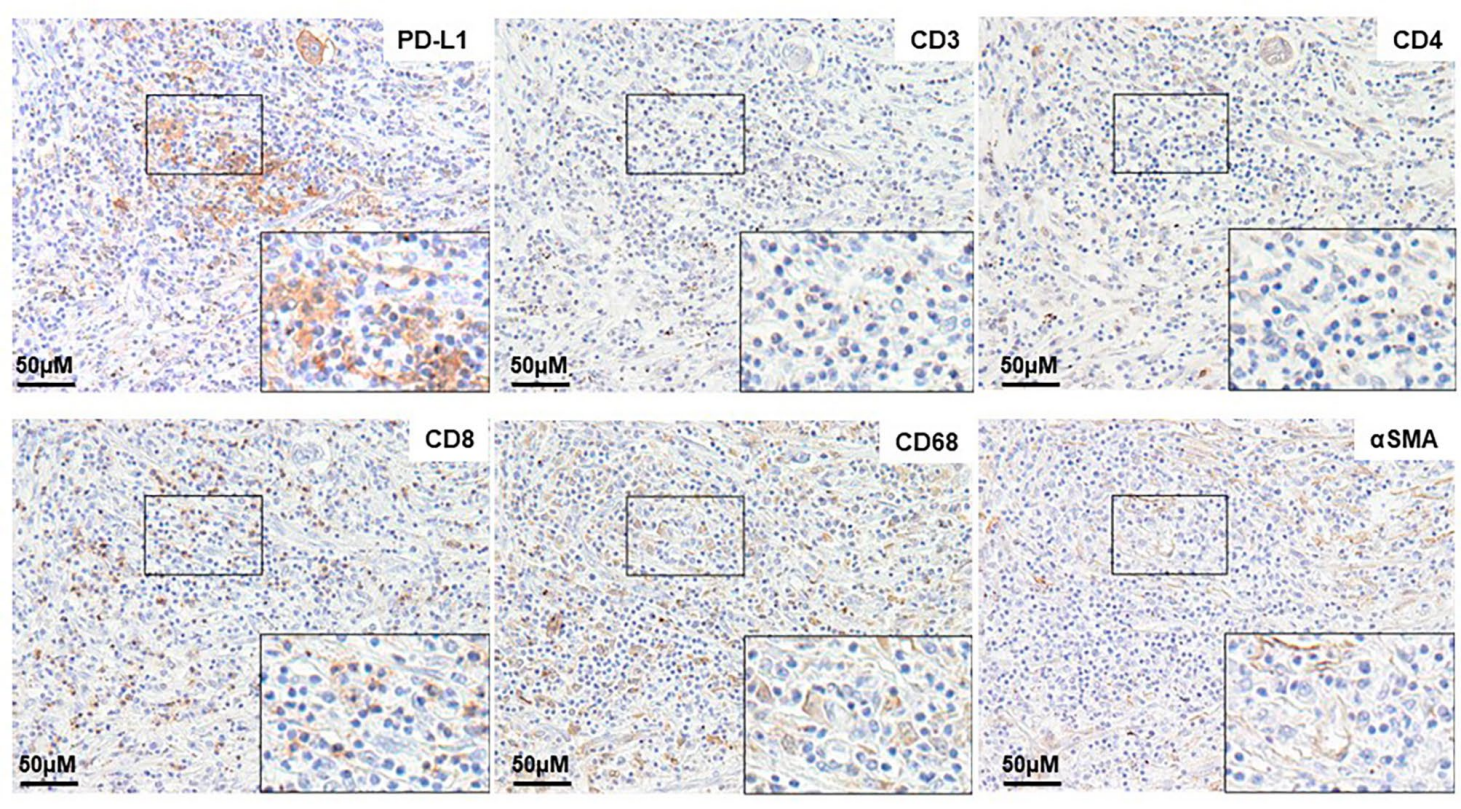
Stromal PD-L1 expression in CRLM. (**a**) Positive stromal PD-L1 expression in mononuclear cells. (**b**) CD3. (**c**) CD4. (**d**) CD8. (**e**) CD68. (**f**) αSMA Magnification of the boxed area (black) is shown in the insets

PD-L1 was mainly expressed by round mononuclear cells in the stroma (Fig. 1a), indicating that most positive cells were lymphocytes. There were few PD-L1-positive cells, which appeared large and round, suggesting macrophages. PD-L1 was infrequently expressed in fibroblasts that were shaped as spindles. To reveal the characteristics of stromal PD-L1-positive cells, immunohistochemistry was performed in serial sections using CD3, CD4, and CD8 as T cell markers, CD68 as a macrophage marker, and αSMA as a fibroblast marker (Fig. 2). PD-L1-positive cells were mainly positive for CD8 and some were positive for CD68.

### Correlations between stromal PD-L1 expression and clinicopathological characteristics

Clinicopathological characteristics in accordance with stromal PD-L1 expression are shown in Table 1. Stromal PD-L1 expression in CRLM tended to associate with a lower tumor grade (*p =* 0.08). Furthermore, there were significantly fewer instances of non-optimally resectable metastases in stromal PD-L1 + patients compared with stromal PD-L1– patients (*p =* 0.04). Primary tumor characteristics did not significantly differ in accordance with stromal PD-L1 expression. Stromal PD-L1 expression was significantly correlated to tumor-specific PD-L1 expression in CRLM (*p =* 0.05).

**Table 1:** Clinicopathological characteristics in accordance with stromal PD-L1 expression

Variables	Stromal PD-L1(-) (*n* = 40)	Stromal PD-L1(+) (*n* = 44)	*p*-value
< **Metastatic tumor characteristics** >			
Age (years)	66.6 ± 12.2	65.1 ± 10.4	0.54
Sex (men / women)	30/10	27/17	0.17
Tumor maximum size (cm)	3.5 ± 2.2	3.6 ± 2.0	0.67
Tumor number (< 5 / ≥5)	25/15	32/12	0.33
H-stage (H1 / H2, 3)	23/17	31/13	0.21
Grade (A / B,C)	17/23	27/17	0.08
Metastasis period (synch / meta)	14/26	24/20	0.07
Pre-operative chemotherapy (- / +)	36/4	37/7	0.42
Post-operative chemotherapy (- / +)	10/30	15/29	0.36
Not optimally resectable (- / +)	9/31	19/25	0.04
Tumor PD-L1 expression (- / +)	21/19	14/30	0.05
PD-1 expression (- / +)	31/9	33/11	0.79
HGP (replacement / desmoplastic / pushing)	20/8/12	10/26/8	0.0009
Replacement subtype (- : +)	20/20	34/10	0.008
Desmoplastic subtype (- : +)	32/8	18/26	0.0002
Pushing subtype (- : +)	28/12	36/8	0.20
TILs (low : high)	35/5	33/11	0.14
**< Primary tumor characteristics >**			
Tumor differentiation (diff./undiff.)	38/2	43/1	0.67
T (2,3/4)*	34/9	30/8	0.99
Location (colon/rectum)	25/15	20/24	0.12
Lymph node metastasis (-/+)	18/22	20/24	0.97
Venous invasion (-/+)*	15/22	15/26	0.72
Lymphatic invasion (-/+)*	12/25	16/25	0.54

Synch/meta: synchronous/metachronous; diff./undiff.: differentiated histological type/undifferentiated histological type; HGP: histopathological growth pattern

*Data for certain patients were unavailable

### Correlation between stromal PD-L1 expression and histological findings

**Fig. 3:**
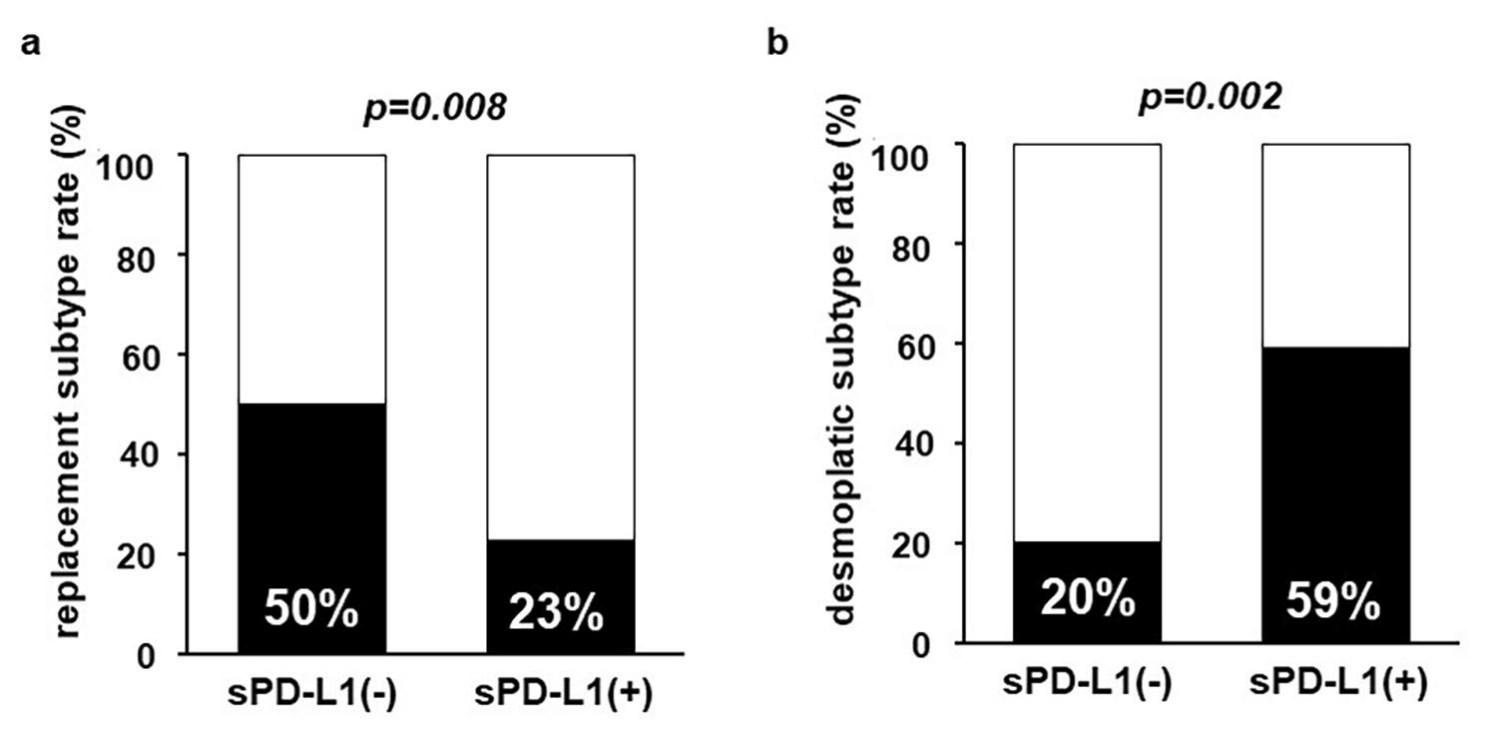
Correlation Between stromal PD-L1 and histological growth patterns. Correlation between stromal PD-L1 expression and the (**a**) replacement subtype and (**b**) desmoplastic type

Stromal PD-L1 expression tended to correlate to a high number of TILs (Table 1, p *=* 0.14), although there was no significant difference. There was no correlation between tumor PD-L1 expression and TILs (*p =* 0.34). Interestingly, stromal PD-L1 expression was associated with the histological growth pattern of CRLM (*p =* 0.0009, Table 1). Stromal PD-L1 expression was inversely related to the replacement subtype (Fig. 3a, *p =* 0.008) and positively related to the desmoplastic type (Fig. 3b, *p =* 0.0002). Clinicopathological characteristics in accordance with the histological growth pattern are shown in S1 Table (replacement subtype) and S2 Table (desmoplastic subtype). The characteristics did not significantly differ in accordance with the histological growth pattern except for stromal PD-L1 expression.

### Influence of stromal PD-L1 expression on survival

**Fig. 4:**
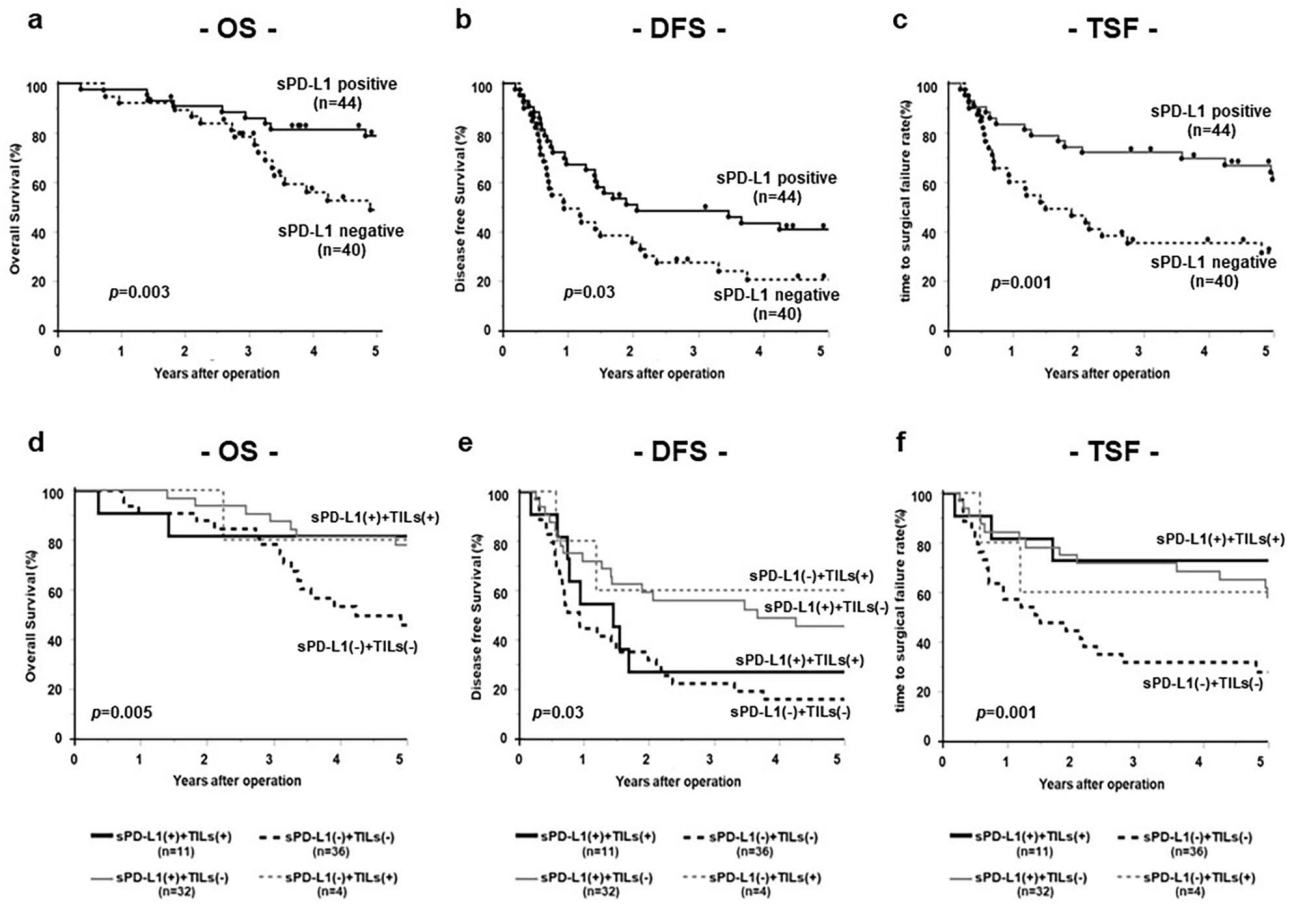
Prognostic value of stromal PD-L1 expression in CRLM Kaplan–Meier survival analysis of CRLM patients with stromal PD-L1 expression: (**a**) Overall survival. (**b**) Disease-free survival. (**c**) Time to surgical failure. Kaplan–Meier survival analysis of CRLM patients with stromal PD-L1 expression combined with TILs: (**d**) Overall survival. (**e**) Disease-free survival. (**f**) Time to surgical failure

The 5-year OS rate of the stromal PD-L1 + group was significantly higher than that of the stromal PD-L1 − group (78.8% vs. 48.8%, *p =* 0.003) (Fig. 4a), and the 5-year DFS rate of the stromal PD-L1 + group was significantly higher than that of the stromal PD-L1 − group (40.9% vs. 20.5%, *p =* 0.03) (Fig. 4b). Furthermore, the 5-year survival rate in accordance with TSF was significantly longer in the stromal PD-L1 + group than in the stromal PD-L1 − group (61.2% vs. 26.9%, *p =* 0.001) (Fig. 4c).

When combined with stromal PD-L1 expression and TILs, stromal PD-L1– and low TIL groups had shorter OS than stromal PD-L1 + and high TIL groups (46.6% vs. 81.8%, *p =* 0.05) (Fig. 4d), and significantly shorter TSF (29.6% vs. 72.7%, *p =* 0.02) (Fig. 4f). PD-L1– and high-TIL groups tended to have longer DFS than stromal PD-L1– and low TIL groups, although it did not show a significant difference (*p =* 0.13, Fig. 4e).

Univariate analysis of OS is shown in Table 2. H1 stage (*p =* 0.03), metastatic grade A (*p =* 0.002), stromal PD-L1 expression in CRLM (*p =* 0.003), tumor PD-L1 expression in CRLM (*p =* 0.004), better differentiation in the primary site (*p =* 0.001), and shallow tumor invasion in the primary site (*p =* 0.04) were significant prognostic factors for longer OS. Multivariate analysis revealed that stromal PD-L1 expression in CRLM [hazard ratio (HR): 0.34, 95% CI 0.15–0.750, *p =* 0.008) and differentiation (HR: 0.02, 95% CI 0.002–0.21, *p =* 0.001) were independent prognostic indicators.

Univariate analysis of DFS is shown in Table 3. Metastatic grade A (*p =* 0.007), synchronous metastasis (*p =* 0.02), stromal PD-L1 expression in CRLM (*p =* 0.03), desmoplastic subtype (*p =* 0.04), and better differentiation in the primary site (*p <* 0.0001) were significant prognostic factors for longer DFS. Multivariate analysis revealed that better differentiation (HR: 0.11, 95% CI 0.03–0.41, *p =* 0.001) was an independent prognostic indicator of DFS.

**Table 2:** Univariate and multivariate analyses of clinicopathological factors associated with overall survival after hepatectomy

Variable	5-year OS rate (%)	Uni-variate *P*-value	Multivariate analysis	
			HR (95% CI)	*P*-value
< **Metastatic tumor characteristics** >				
Age (< 70 years / ≥70 years)	74.3 / 59.1	0.31		
Sex (men/women)	64.3 / 70.6	0.72		
Tumor maximum size (< 5 cm / ≥5 cm)	67.5 / 60.7	0.08		
Tumor number (< 5 / ≥5)	70.1 / 51.8	0.35		
H-stage (H1/H2, 3)	74.3 / 48.4	0.03	0.81 (0.28–2.30)	0.69
Grade (A/B,C)	81.4 / 48.1	0.002	0.43 (0.15–1.22)	0.11
Metastasis period (synch/meta)	80.3 / 52.7	0.06		
Pre-operative chemotherapy (- : +)	65.5 / 6757	0.51		
Post-operative chemotherapy (- : +)	61.9 / 67.7	0.52		
Stromal PD-L1 (- : +)	48.8 / 78.8	0.003	0.33 (0.15–0.75)	0.008
Tumor PD-L1 (- : +)	51.1 / 87.5	0.004	0.65 (0.30–1.40)	0.27
PD-1 (- : +)	60.2 / 83.8	0.05		
TILs (low : high)	62.6 / 81.3	0.15		
Replacement subtype (- : +)	68.5 / 60.4	0.71		
Desmoplastic subtype (- : +)	55.8 / 75.8	0.05		
Pushing subtype (- : +)	70.9 / 47.0	0.05		
**< Primary tumor characteristics >**				
Colon / rectum	65.4 / 66.4	0.63		
Tumor differentiation (diff./undiff.)	67.9 / 00.0	< 0.001	0.02 (0.001–0.32)	0.001
T (2,3/4)	74.0 / 35.2	0.04	0.57 (0.25–1.32)	0.19
Lymph node metastasis (- : +)	74.1 / 58.8	0.14		
Lymphatic invasion (- : +)	74.1 / 61.5	0.69		
Venous invasion (- : +)	77.7 / 58.3	0.24		

Synch/meta: synchronous/metachronous; diff./undiff.: differentiated histological type/undifferentiated histological type

**Table 3:** Univariate and multivariate analyses of clinicopathological factors associated with disease-free survival after hepatectomy

Variables	5-year DFS rate (%)	Uni-variate *P*-value	Multivariate analysis	
			HR (95% CI)	*P*-value
< **Metastatic tumor characteristics** >				
Age (< 70 years / ≥70 years)	27.3 / 35.3	0.83		
Sex (men/women)	34.3 / 27.1	0.86		
Tumor maximum size (< 5 cm / ≥5 cm)	33.5 / 26.6	0.41		
Tumor number (< 5 / ≥5)	33.9 / 32.0	0.31		
H-stage (H1/H2, 3)	36.8 / 21.4	0.08		
Grade (A/B,C)	41.1 / 18.4	0.007	0.57 (0.32–1.01)	0.05
Metastasis period (synch/meta)	41.7 / 23.0	0.02	0.69 (0.38–1.23)	0.21
Pre-operative chemotherapy (- : +)	31.8 / 30.7	0.64		
Post-operative chemotherapy (- : +)	39.2 / 28.7	0.44		
Stromal PD-L1 (- : +)	20.5 / 41.0	0.03	0.71 (0.38–1.32)	0.28
Tumor PD-L1 (- : +)	26.6 / 35.8	0.39		
PD-1 (- : +)	25.5 / 52.8	0.08		
TILs (low : high)	30.6 / 37.5	0.69		
Replacement subtype (- : +)	34.8 / 25.2	0.42		
Desmoplastic subtype (- : +)	21.5 / 45.6	0.04	0.75 (0.39–1.44)	0.39
Pushing subtype (- : +)	36.5 / 15.9	0.22		
**< Primary tumor characteristics >**				
Colon / rectum	37.9 / 24.2	0.40		
Tumor differentiation (diff./undiff.)	30.6 / 00.0	< 0.0001	0.11 (0.03–0.41)	0.001
T (2,3/4)	31.8 / 23.4	0.46		
Lymph node metastasis (- : +)	42.0 / 22.5	0.09		
Lymphatic invasion (- : +)	24.1 / 35.8	0.34		
Venous invasion (- : +)	31.8 / 23.4	0.56		

Synch/meta: synchronous/metachronous; diff./undiff.: differentiated histological type/undifferentiated histological type

## Discussion

Evidence indicates that the tumor stroma, which comprises the tumor microenvironment, is required for tumor growth and progression [32]. In the host microenvironment, the liver contains phenotypically distinct stromal cells including macrophages, fibroblasts, lymphocytes, and dendritic cells. These cells interact in a complex manner mediated by cytokines and chemokines [33]. The tumor stroma promotes tumor cell proliferation and dissemination through various mechanisms. Tumor stroma remodels the extracellular matrix and recruits inflammatory cells [34, 35]. Furthermore, the tumor stroma has been implicated in the prognostic outcomes of CRC patients [35]. However, the prognostic value of stromal PD-L1 expression in CRC [16, 17] and tumor PD-L1 expression [6, 36] is highly debated. Only one study reported the stromal PD-L1 expression in colorectal liver oligometastasis [20]. Unlike our study results, PD-L1 expression was correlate to poor prognosis in patients with liver oligometastasis.

Because PD-1/PD-L1 signaling attenuates host immunity and maintains peripheral tolerance [37], the association of the immunosuppressive ligand PD-L1 with a better prognosis of CRLM may seem counterintuitive. This apparent contradiction may be explained if stromal PD-L1 expression acts as an adaptive anti-tumor response to tumor antigens mediated by an activated immune escape pathway. This possibility is consistent with our findings that stromal PD-L1 expression tended to correlate to a high number of TILs. For example, CRLM patients with CD8 + TILs had better OS than patients with CD8- TILs [38, 39]. Furthermore, a high CD8+/CD4 + ratio and low FOXP3/CD8 ratio correlate to longer survival of stromal PD-L1 + patients, but not that of stromal PD-L1 − patients with esophageal cancer [40]. These findings support the conclusion that immune-mediated and tumor-intrinsic oncogenic activation controls stromal PD-L1 expression. Furthermore, patients with PD-L1+/TILs + have longer OS and TSF but shorter DFS in this study. One possible reason could be a tolerability of chemotherapy. The previous report showed TILs were negatively associated with frailty [41]. The frailty increased the risk of chemotherapy. In this point, the patients with PD-L1+/TILs + could show the good tolerance for treatment without frail. Future analysis will be required to determine the impact of frailty on the survival in patients with PD-L1+/TILs+.

Here, we found that stromal PD-L1 was mainly expressed by round mononuclear cells in the stroma, indicating lymphocytes as its source. Representative images of IHC staining revealed PD-L1-positive cells were mainly showed positive for CD8. Conversely, there were few PD-L1-positive cells showed positive for CD3, CD4 and αSMA. TIL subsets, including CD19, CD20, and FOXP3, should be investigated and the balance of TILs between immune-reactive and immune-tolerant should be determined in future studies. In this study, stromal PD-L1 expression was correlated to a stronger influence on the prognosis of CRLM compared with tumor PD-L1 expression and the number of TILs. Moreover, CRLM patients with stromal PD-L1- and low TILs had the lowest OS and TSF rates. Furthermore, PD-L1- and high TIL groups tended to have longer DFS than stromal PD-L1– and low TIL groups. TILs may contribute to the different characteristics of stromal PD-L1-positive cells. Therefore, it will be necessary to simultaneously evaluate PD-L1 expression in tumors and stromal cells as well as the proportion of TIL subsets in CRLM. Furthermore, recent study revealed that peripheral PD-1/PD-L1 expression in circulating T lymphocytes had a significant consistency with PD-L1 expression in immune cells in breast cancer [42]. For the clinical application, the relationship between stromal PD-L1 and PD-L1 expression in circulating T lymphocytes should be investigated in future study. It could provide an alternative choice of tissue biopsy to detect the stromal PD-L1 expression for the patients with CRLM.

In this study, we also found that stromal PD-L1 expression in CRLM was inversely related to the replacement subtype and indicated a better prognosis. The main histological characteristics of tumor growth in CRC include pushing, replacement, and desmoplastic [43]. Patients with the replacement subtype have a worse prognosis after curative liver resection compared with patients with the desmoplastic subtype [44, 45]. The different histological patterns of CRLM are associated with different types of tumor vascularization [43]. The replacement subtype shows a non-angiogenic growth pattern in contrast to the desmoplastic subtype [46]. Because vascular co-option from the normal liver is highly efficient, the replacement subtype shows minimal hypoxia [47]. Furthermore, angiotropism resembling the co-opted capillary bed contributes cancer cell motility and invasion during replacement growth [48]. This may be a reason that tumors with the replacement subtype show aggressive characteristics resulting in a poor prognosis. The other reason for the difference between replacement and desmoplastic subtypes may be the immune response to metastatic tumors. A previous study revealed that CD8 + TILs in desmoplastic subtypes are associated with longer survival [49]. Furthermore, a tumor with the replacement subtype has reduced infiltration of CD8 + immune cells [50]. In this study, stromal PD-L1 expression tended to correlate to high TILs, although it was not significantly different. Taken together, these findings suggest that patients with stromal PD-L1 expression and a favorable prognosis have the ability to perform less replacement growth, possibly through immune infiltration. Thus, to confirm and clarify this association, further studies are required to delineate the role and influence of stromal cells to the histological characteristics of tumor growth.

This study has certain limitations. Firstly, this was a retrospective single-center study of a limited number of patients. The correlation between TILs and stromal PD-L1 will be evident with cohort study. Moreover, future research should analyze TIL subsets and the histological characteristics of tumor growth. Secondly, this study employed immunohistochemistry as the only method to evaluate protein expression in CRLM. Our result should be confirmed through a prospective study by flow cytometry analysis.

## Conclusions

Our present study demonstrates the strong influence of stromal PD-L1 expression on the prognostic outcomes of CRLM patients. The expression of stromal PD-L1 served as a better independent prognostic factor for OS. Patients with stromal PD-L1-negative expression and low TILs had the worst OS. These results contribute to a better understanding of the interactions between a tumor and its microenvironment, and will thereby enable prediction of the prognoses of CRLM patients.

### Electronic supplementary material

Below is the link to the electronic supplementary material.

Supplementary Material 1

## Data Availability

The datasets analyzed during the current study are available from the corresponding author on reasonable request.
