# Supplementary material for: Role of stromal PD-L1 expression in colorectal liver metastasis
Source: BMC Cancer. 2024 Jan 17;24:97. doi: 10.1186/s12885-024-11869-8 (PMC10795256; doi:10.1186/s12885-024-11869-8)
Supplement: Supplementary file 1 — Supplementary Material 1 [file 12885_2024_11869_MOESM1_ESM.doc]

S1 Table. Clinicopathological characteristics in accordance with replacement subtype

| **Variables** | **Replacement subtype (-)**  **(n=54)** | **Replacement subtype (+) (n=30)** | **p-value** |
| --- | --- | --- | --- |
| **< Metastatic tumor characteristics >** |  |  |  |
| Age (years) | 64.2 ± 11.4 | 68.6 ± 10.6 | 0.19 |
| Sex (men / women) | 36/18 | 21/9 | 0.75 |
| Tumor maximum size (cm) | 3.9 ± 2.3 | 3.0 ± 1.5 | 0.15 |
| Tumor number (<5 / ≥5 ) | 35/19 | 22/8 | 0.42 |
| H-stage (H1 / H2, 3) | 34/20 | 20/10 | 0.73 |
| Grade (A / B,C) | 31/23 | 13/17 | 0.22 |
| Metastasis period (synch / meta) | 28/26 | 18/12 | 0.47 |
| Pre-operative chemotherapy (- / +) | 49/5 | 24/6 | 0.17 |
| Post-operative chemotherapy (- / +) | 15/39 | 10/20 | 0.60 |
| Not optimally resectable (- / +) | 19/35 | 9/21 | 0.63 |
| Stromal PD-L1 expression (- / +) | 20/34 | 20/10 | 0.008 |
| Tumor PD-L1 expression (- / +) | 20/34 | 15/15 | 0.25 |
| PD-1 expression (- / +) | 40/14 | 24/6 | 0.54 |
| TILs (low / high) | 42/12 | 26/4 | 0.31 |
| **< Primary tumor characteristics >** |  |  |  |
| Tumor differentiation (diff. / undiff.) | 52/2 | 29/1 | 0.93 |
| T (2,3 / 4)* | 40/11 | 24/5 | 0.64 |
| Location (colon / rectum) | 30/24 | 15/15 | 0.62 |
| Lymph node metastasis (- / +) | 29/25 | 9/21 | 0.35 |
| Venous invasion (- / +)* | 18/31 | 12/17 | 0.68 |
| Lymphatic invasion (- / +)* | 18/31 | 10/19 | 0.84 |

Synch/meta: synchronous/metachronous; diff./undiff.: differentiated histological type/undifferentiated histological type.

*Data for certain patients were unavailable.

S2 Table. Clinicopathological characteristics in accordance with desmoplastic subtype

| **Variables** | **Desmoplastic subtype (-)**  **(n=54)** | **Desmoplastic subtype (+) (n=30)** | **p-value** |
| --- | --- | --- | --- |
| **< Metastatic tumor characteristics >** |  |  |  |
| Age (years) | 65.9 ± 12.3 | 65.7 ± 9.5 | 0.84 |
| Sex (men / women) | 32/18 | 25/9 | 0.36 |
| Tumor maximum size (cm) | 3.3 ± 1.8 | 4.0 ± 2.3 | 0.45 |
| Tumor number (<5 / ≥5 ) | 33/17 | 24/10 | 0.66 |
| H-stage (H1 / H2, 3) | 32/18 | 22/12 | 0.94 |
| Grade (A / B,C) | 23/27 | 21/13 | 0.15 |
| Metastasis period (synch / meta) | 31/19 | 15/19 | 0.11 |
| Pre-operative chemotherapy (- / +) | 42/8 | 31/3 | 0.33 |
| Post-operative chemotherapy (- / +) | 13/37 | 12/22 | 0.36 |
| Not optimally resectable (- / +) | 14/36 | 14/20 | 0.21 |
| Stromal PD-L1 expression (- / +) | 32/18 | 8/26 | 0.0002 |
| Tumor PD-L1 expression (- / +) | 25/25 | 10/24 | 0.06 |
| PD-1 expression (- / +) | 41/9 | 23/11 | 0.13 |
| TILs (low / high) | 41/9 | 27/7 | 0.77 |
| **< Primary tumor characteristics >** |  |  |  |
| Tumor differentiation (diff. / undiff.) | 48/2 | 33/1 | 0.80 |
| T (2,3 / 4)* | 35/13 | 29/4 | 0.10 |
| Location (colon / rectum) | 23/27 | 22/12 | 0.09 |
| Lymph node metastasis (- / +) | 19/31 | 19/15 | 0.11 |
| Venous invasion (- / +)* | 18/28 | 12/20 | 0.88 |
| Lymphatic invasion (- / +)* | 14/32 | 14/18 | 0.23 |

Synch/meta: synchronous/metachronous; diff./undiff.: differentiated histological type/undifferentiated histological type.

*Data for certain patients were unavailable.
